# *Mycoplasma genitalium*: An Overlooked Sexually Transmitted Pathogen in Women?

**DOI:** 10.1155/2016/4513089

**Published:** 2016-04-24

**Authors:** Samsiya Ona, Rose L. Molina, Khady Diouf

**Affiliations:** ^1^Department of Obstetrics and Gynecology, Brigham and Women's Hospital, 75 Francis Street, Boston, MA 02115, USA; ^2^Department of Obstetrics and Gynecology, Beth Israel Deaconess Medical Center, 330 Brookline Avenue, Boston, MA 02215, USA; ^3^Division of Women's Health, Brigham and Women's Hospital, 1620 Tremont Street, OBC-34, Boston, MA 02120, USA

## Abstract

*Mycoplasma genitalium* is a facultative anaerobic organism and a recognized cause of nongonococcal urethritis in men. In women,* M. genitalium* has been associated with cervicitis, endometritis, pelvic inflammatory disease (PID), infertility, susceptibility to human immunodeficiency virus (HIV), and adverse birth outcomes, indicating a consistent relationship with female genital tract pathology. The global prevalence of* M. genitalium* among symptomatic and asymptomatic sexually active women ranges between 1 and 6.4%.* M. genitalium* may play a role in pathogenesis as an independent sexually transmitted pathogen or by facilitating coinfection with another pathogen. The long-term reproductive consequences of* M. genitalium* infection in asymptomatic individuals need to be investigated further. Though screening for this pathogen is not currently recommended, it should be considered in high-risk populations. Recent guidelines from the Centers for Disease Control regarding first-line treatment for PID do not cover* M. genitalium* but recommend considering treatment in patients without improvement on standard PID regimens. Prospective studies on the prevalence, pathophysiology, and long-term reproductive consequences of* M. genitalium* infection in the general population are needed to determine if screening protocols are necessary. New treatment regimens need to be investigated due to increasing drug resistance.

## 1. Introduction

*M. genitalium* was first identified in 1980 from the urethral specimens of two men with nongonococcal urethritis (NGU) [1, 2]. Its prevalence in men presenting with urethritis is between 30 and 40% [1, 3]. Further, the presence of* M. genitalium* in men is associated with a 5.5-fold increased risk of NGU [4, 5]. In women,* M. genitalium* has been linked to cervicitis, endometritis, pelvic inflammatory disease (PID), infertility, human immunodeficiency virus (HIV), and adverse birth outcomes [1]. In the United States, the prevalence of* M. genitalium* among women is thought to be around 1%, slightly higher than* Neisseria gonorrhoeae* prevalence (0.4%) and less than* Chlamydia trachomatis* prevalence (3%) based on a nationally representative sample of young adults [3]. Despite initial contradictory findings regarding the association between* M. genitalium* and female genital tract pathology [6, 7], several studies have since confirmed this association [1, 3, 8], which has recently been reviewed in a meta-analysis [9]. The development of nucleic acid amplification in the 1990s has facilitated several epidemiologic studies that have examined the association of* M. genitalium* with PID [1, 10, 11]. In the 2015 sexually transmitted infection (STI) treatment guidelines, the Centers for Disease Control (CDC) calls attention to* M. genitalium* as an emerging sexually transmitted pathogen in women [12].

PID is a polymicrobial disease commonly diagnosed in women of reproductive age [13]. The prevalence among women aged 15–44 in the United States declined since 1995 from ~8.6% down to ~5.7% in 2002 and leveled off between 2006 and 2010 at 5% [14]. Although a third to half of PID cases have been associated with* N. gonorrhoeae* and/or* C. trachomatis*, many cases have an unknown etiology [12]. It is well known that PID can lead to serious reproductive problems including infertility, chronic pelvic pain, ectopic pregnancy, and recurrent infections. The independent association between* M. genitalium* and PID confirmed by several studies [15] raises concern that* M. genitalium* may play a pathogenic role, particularly in cases where other STIs are not identified. Thus, there is a need for PID treatment regimens to cover* M. genitalium*. Further complicating management, several studies have now identified* M. genitalium* treatment resistance among infected women [15–18].

Most studies on* M. genitalium* are observational studies of variable sample sizes, some very small, with very few randomized trials. Consequently, some of the study findings may not be transferable to most populations. The prevalence of* M. genitalium* from cohort studies done in high-risk populations is considerably higher than the prevalence found in the general population [1, 2].* M. genitalium* has also been described in sexually abstinent women, putting into question the criteria for screening for this pathogen.

Our objective is to review the evidence in the literature regarding the association of* M. genitalium* with genital tract pathology in women and to identify needed areas of research regarding the pathophysiology, clinical manifestations, screening, and treatment of this pathogen.

## 2. Materials and Methods

An initial PubMed search was conducted using the terms “*Mycoplasma genitalium,*” “*Mycoplasma genitalium* women,” and “prevalence of* Mycoplasma genitalium* in asymptomatic women,” which identified 1064 articles. Articles were excluded for lack of relevance by reviewing titles, abstracts, and content. English articles presenting relevant data stratified by sex and conducted exclusively in women were included. As a result, 66 articles were included in this review.

## 3. Results and Discussion

### 3.1. Clinical Updates on* M. genitalium* as a Sexually Transmitted Infection in Women

#### 3.1.1. Epidemiology

*M. genitalium* is one of the most common microorganisms associated with genital tract infections and is increasingly recognized as a STI [1, 19–21].* M. genitalium* infection has several clinical features consistent with sexual transmission, including higher detection among sexually active individuals compared to sexually naïve adolescents, detection in partners of infected individuals, and predominance in younger individuals with multiple sexual partners and men who have sex with men (particularly those infected with HIV) [1, 3, 8, 19, 22, 23]. Several studies that had identified* Mycoplasma* as a STI have showed statistically significant increased rates of infection among sexually active women, with rate/risk of infection increasing with 2 or more sexual partners. One study reported that the prevalence of* M. genitalium* increases by 10% with each additional sexual partner [3]. It has also been shown that women with infected partners are also at increased risk [3] so sexual activity in itself appears to be a major risk factor but is not the only determinant factor for infection. Further, several studies have shown an independent association between* M. genitalium* and genital infection. It however appears that not all carriers are symptomatic as evidenced by general population studies [24].

Several clinical associations with* M. genitalium* infection have been identified. In one prospective study,* M. genitalium* was found most frequently among women aged ≤24years, those with a history of abortion, and those with first intercourse after 20 years [35]. This last association seems counterintuitive but may be related to a higher chance of clearing the infection when women are first exposed to the organism at a younger age, although there are no studies to date to support this argument. Overall, most evidence suggests a low prevalence of* M. genitalium* among asymptomatic women [25], which may make screening efforts low-yield [21, 25, 28, 35, 36].

Most of the epidemiological studies on* Mycoplasma* infection have been conducted in high-risk populations, such as symptomatic and asymptomatic patients attending STI clinics. This introduces a sampling bias and limits the conclusions regarding* M. genitalium* as an independent STI in the general population [25, 36].

Table 1 summarizes the studies regarding the prevalence of* M. genitalium*. The prevalence in the general population is not known since routine screening is not done but some studies have estimated the global prevalence of* M. genitalium* among women to range between 1 and 6.4% [37–39]. Prevalence studies have usually included women attending STI clinics or those infected with HIV. Clarivet et al. found a low rate of 0.1% in asymptomatic women [25] whereas Gaydos et al. found a rate close to 20% among women attending STI clinic in Baltimore (~70% of these women were symptomatic) [2]. Studies from adolescent clinics, STI clinics, and emergency departments in the United States have identified* M. genitalium* as a genital tract microorganism in 15–20% of young women reporting genitourinary symptoms or at risk for STIs based on clinical history [13].

*M. genitalium* coinfection with* C. trachomatis* has also been recognized. In a cross-sectional case-control study, 4.5% of asymptomatic patients were found to be positive for* M. genitalium* [27], and ~5% of individuals infected with* C. trachomatis* were coinfected with* M. genitalium* [27]. Asymptomatic study participants, usually recruited from a convenience group of STI clinic attendees reported no genital tract symptoms [21]. The prevalence was higher among younger women 18–24 years of age compared to older women (7.9% for* C. trachomatis* and 2.4% for* M. genitalium*, resp.) [35].

#### 3.1.2. Clinical Manifestations

*M. genitalium* has been associated with typical PID symptoms such as pelvic pain, abnormal vaginal discharge, fever, nausea, and vomiting. Symptomatic women who are positive for* M. genitalium* are more likely to report postcoital bleeding, which could be due to cervicitis, compared to women negative for the organism (AOR 5.8; 95% CI 1.4–23.3, after adjusting for age and coinfections) [36]. Most* M. genitalium* infections are asymptomatic in women [24] and roughly half of women (56.2%) who test positive for the organism are asymptomatic [37]. Like* C. trachomatis, M. genitalium* can lead to “silent” PID infections with mild symptoms relative to* N. gonorrhoeae* associated PID symptoms [13]. Bjartling et al. found comparable rates of abnormal vaginal wet smear, cervical friability or tenderness, fever, and level of serum C-reactive protein (CRP) between* M. genitalium*-positive women and negative controls [27]. However,* M. genitalium*-positive women were more likely to report postcoital bleeding than the negative controls [AOR 2.00 (1.10–3.61)]. Further, women with* M. genitalium* were more likely to have combined cervical tenderness, postcoital bleeding, and abnormal vaginal discharge [AOR 2.71 (1.50–4.90)] compared to women not infected with* M. genitalium* [27].

#### 3.1.3. *M*.* genitalium*: An Emerging Cause of Pelvic Inflammatory Disease (PID)

PID is an inflammatory disease that can include one or more of the following conditions: endometritis, salpingitis, tuboovarian abscess, and pelvic peritonitis. PID is described as a polymicrobial syndrome, mainly caused by anaerobic bacterial species [1].* N. gonorrhoeae* and* C. trachomatis* are the most commonly diagnosed organisms in PID, yet up to 70% of cases are of indeterminate etiology [1, 40]. Organisms of the vaginal flora such as* Mycoplasma*,* Ureaplasma, Gardnerella vaginalis, Escherichia coli,* and anaerobes have also been associated with PID. With the development of nucleic acid amplification tests (NAATs), the incidence of biopsy-proven endometritis or clinical PID associated with* M. genitalium* has increased [1, 27]. Women positive for* M. genitalium* were found to be twice as likely to have histology-proven endometritis than women testing negative after adjusting for age, race,* N. gonorrhoeae,* and* C. trachomatis* [AOR 2.0 (1.0 to 4.2)] [15, 41]. Despite being less studied, postabortal PID has been shown to be strongly associated with* M. genitalium* [AOR 6.3 (1.6–25.3)] [1, 13]. Several cross-sectional studies have investigated the independent association between* M. genitalium* and PID. For example, one prospective study reported a thirteenfold increased incidence of endometritis in the presence of* M. genitalium* at 30-day follow-up visits among an urban population of women in the United States with clinical PID without concurrent* N. gonorrhoeae* and* C. trachomatis* infection [15]. A recent meta-analysis shows pooled odds ratios of 1.66 [95% CI, 1.35–2.04] for cervicitis and 2.43 for infertility [95% CI, .93–6.34] among* M. genitalium* infected women [9].

#### 3.1.4. *Mycoplasma genitalium* and Its Association with Other STIs and Malignancies

*M. genitalium* has been associated with increased susceptibility to HIV infection [42, 43]. Unlike most other* Mycoplasma* species,* M. genitalium* can attach to the surface of epithelial cells and invade the cells with a specialized tip structure [4]. In an* in vitro* model, Das et al. showed that* M. genitalium* increased the risk of HIV infection by infecting the epithelial layer, reducing its integrity, and activating HIV cell targets beyond the epithelial layer, thereby promoting transmission and reproduction within the host and increasing viral shedding through mucosal surfaces [42]. Vandepitte et al. in their nested case-control study found evidence of a temporal relationship between* M. genitalium* and HIV acquisition [43]. The association was only found among the subgroup that was tested for* M. genitalium* three months prior to first HIV-positive results compared to the group with earlier HIV testing (aOR = 7.19; 95% CI 1.68 to 30.77) [43]. Further studies have shown a positive association between* M. genitalium* and high-risk human papilloma virus (HR-HPV) infection. For example, one study of female sex workers showed that 39.6% were positive for* M. genitalium* and HR-HPV [29]. In addition, Zarei et al. have demonstrated an association between chronic* M. genitalium* infection and ovarian cancer and lymphoma [44]. However, these studies did not control for the sexual behavior of women and their partners, limiting the generalizability of the results.

#### 3.1.5. Mycoplasma in Pregnancy

All* Mycoplasma* species have been associated with perinatal morbidity and mortality [45]. A US-based cohort study demonstrated a 2.5-fold increase in preterm birth in women with* M. genitalium* infection who presented with contractions between 23 and 32 weeks of gestation compared to noninfected women (AOR 2.5; 95% CI 1.2–6.0) [1]. In a meta-analysis of six studies,* M. genitalium* was associated with preterm birth with a pooled OR of 1.89 (95% CI, 1.25–2.85) and also associated with spontaneous abortion with a pooled OR of 1.82 (95% CI, 1.10–3.03) [9].

Of the* Mycoplasmas*,* M. hominis* and* Ureaplasma* have been most associated with chorioamnionitis and are thought to contribute to these adverse effects [45]. While* M. hominis* has not been associated with PID, it has been associated with upper respiratory infections, nervous system infections, neonatal bacteremia, and meningoencephalitis, unlike* M. genitalium* [46].

#### 3.1.6. Diagnosis and Screening

*Mycoplasma Diagnosis*.* M. genitalium* is a small bacterium of the Mollicutes class with no cell wall and a genome of only 580 kilobases in size [1, 47]. Consequently, it cannot be detected by gram stain and is extremely difficult to culture requiring up to 6 months for growth [12]. Its genome is most similar to* Mycoplasma pneumonia* [48], which causes atypical bacterial pneumonia. Currently there is no FDA-approved diagnostic test for* M. genitalium* [1]. Given the difficulty with culturing the organism and the lack of standardized serological tests for* M. genitalium,* NAATs in the form of polymerase chain reaction (PCR) assays are almost exclusively carried out for the diagnosis of* M. genitalium* in the research setting. Some PCR assays have demonstrated >95% specificity and sensitivity [49]. A recent study reported loop-mediated isothermal amplification (LAMP) as a novel NAAT, which has similar sensitivity to a PCR assay [50].

To date four types of specimens can be collected for the detection of* M. genitalium*: vaginal swab, first void urine, and endocervical and rectal swabs. Some studies in the United States have shown that NAATs with vaginal swab specimens have the highest relative sensitivity compared to urine and endocervical specimens [10, 51]. Further, self-obtained vaginal swabs have been found to yield similar test sensitivities to clinician-obtained specimens [10]. In an earlier study conducted in Seattle, WA, among symptomatic women attending a STI clinic, the specimen with the highest sensitivities was the vaginal specimen PCR: reported sensitivities were 91%, 53%, and 65% for vaginal, cervical, and urine specimens, respectively [51]. In a subsequent cross-sectional study among women attending a STI clinic in New Orleans, the relative sensitivity of PCR was 85.7% for the vaginal swab specimen, 74.3% for the endocervical swab specimen, 61.4% for the urine specimen, and 24.3% for the rectal swab specimen for the detection of* M. genitalium* in women [10]. Consequently, vaginal swabs are currently the most commonly used specimens for detecting* M. genitalium* through PCR.

*To Screen or Not to Screen*. The 2015 CDC sexually transmitted disease treatment guidelines recommend that all women diagnosed with PID should also be tested for HIV, gonorrhea, and chlamydia [12]. There are no recommendations regarding* M. genitalium* screening given the lack of data around the utility of screening and the lack of a FDA-approved testing modality for commercial use [12].

Given the higher prevalence of* M. genitalium* in high-risk women [1] and its reported association with PID, infertility, and adverse pregnancy outcomes, it would be reasonable to test symptomatic women for* M. genitalium* if NAAT is available. Further, in patients whose symptoms are refractory to appropriate antibiotic therapy for PID, cervicitis, and endometritis, testing for* M. genitalium* may be clinically beneficial and indicated based on current data.

There is ongoing debate regarding possible cost, benefits, and harm of universal screening for* M. genitalium* among asymptomatic patients given that most carriers are likely asymptomatic. Given limited data, this decision should be based on a discussion between providers and patients in the context of personal risk factors, as official screening recommendations will not be made until better quality data on cost, harm, and benefits are available.

### 3.2. Treating* M. genitalium* Infection

Azithromycin and doxycycline are the current first-line treatment for cervicitis and NGU [5]. One of the initial randomized controlled trials on* Mycoplasma genitalium* treatment reported more effective treatment with a single 1 g of azithromycin compared to doxycycline 100 mg BID for 7 days in the USA [52]. Cure rates with azithromycin ranged from 67 to 87% [5]. However, higher treatment failures with single 1 g of azithromycin were reported with a decline in efficacy down to 60% [53, 54] and to 39% in the most recent study [55]. Treatment failure with azithromycin is due to an isolated point mutation on 23 rRNA gene in numerous* M. genitalium* populations [17], with up to 50% of cases reported [12].

Due to these poor efficacy rates, alternative azithromycin regimens have been investigated [5]. Several studies have examined an extended 1.5 g azithromycin (500 mg on day 1, followed by 250 mg daily for 4 days) and a single higher dose of 2 g azithromycin once with the rationale that an extended azithromycin-containing regimen decreases the risk of acquired macrolide resistance when initiated first-line among patients without preexisting macrolide resistance. A similar trend has also been noted with doxycycline [52]. Unfortunately, a recent randomized controlled trial found declining microbiological cure rate for the extended regimen and single 2 g regimen to 25–81% (wide range based on different studies) and 73%, respectively [5].

In light of the rising azithromycin resistance, moxifloxacin had been introduced as a second-line treatment option. Moxifloxacin, a fluoroquinolone, was thought to be a reliable alternative with a reported 100% cure rate initially [30, 56]. According to the 2015 CDC guidelines, women with PID who do not respond to the first-line treatment within 7–10 days should be considered as possibly infected with* M. genitalium* and treated with moxifloxacin 400 mg/day for 14 days [12, 57]. It is not used as first-line due to more significant adverse effects associated with moxifloxacin relative to azithromycin, such as tendon rupture, although these significant adverse effects remain rare. However, as of 2013, increasing treatment failures have also been noted due to bacterial resistance to moxifloxacin with failure rates ranging between 10% and 15% [17, 18, 58]. Given increasing moxifloxacin resistance, monotherapy has the potential to increase the risk of multidrug-resistant strains.

Other fluoroquinolones that have been investigated and proven to remain effective include gatifloxacin and sitafloxacin [59]. Other fluoroquinolones such as gemifloxacin, sparfloxacin, grepafloxacin, trovafloxacin, and garenoxacin have been shown to be effective against* M. genitalium in vitro* but lack human studies [59]. Ciprofloxacin, ofloxacin, and levofloxacin reportedly have poor activity against the microbe relative to moxifloxacin [59]. Pristinamycin is a streptogramin that is used to treat vancomycin-resistant* Enterococcus faecium* bacteremia and complicated skin infections due to MRSA [60]. Treatment of* M. genitalium* with pristinamycin (1 g 6 hourly for 10 days) led to negative PCR results 28 days after treatment [60]. This regimen appears promising for the treatment of multidrug-resistant* M. genitalium* but has not been well studied to inform optimal dosing and is reportedly expensive with limited availability [60].

Given the organism's propensity for drug resistance, follow-up testing to document treatment response is reasonable. Some authors advocate for a test of cure (TOC) in 3-4 weeks after treatment with resistance profiling in those with persistent infection despite treatment [16]. Most studies on TOC have been conducted in men, with fewer studies done in women. A retrospective cohort study performed TOC at 1 month from the initiation of therapy with azithromycin-containing regimens to identify resistant infections [61]. However, a later prospective cohort study investigated the optimal time for TOC and reported negative TOC within an average of 14 days (12–15 days) for infected patients that were susceptible to a single 1 g of azithromycin, which was used as the first-line treatment [55]. Those that appeared resistant were further treated with moxifloxacin 400 mg daily for 10 days with a negative TOC at 28 days for responders [55]. Furthermore, Falk et al. showed that individuals treated with azithromycin had a negative PCR within 8 days and those treated with moxifloxacin had a negative PCR within 1 week [62]. However, it was further discussed that early negative PCR may be related to low DNA levels for detection soon after treatment initiation with resistance detected at 10 days after treatment initiation with azithromycin and eventually recolonization requiring further treatment [62]. Hence it was concluded that optimal timing for the most reliable TOC should take place 3-4 weeks after treatment [62], which correlates with an earlier Japanese study performed among men [63].

Testing and/or empirical treatment of partners within the preceding 60 days of diagnosis are also strongly recommended for women with confirmed positive* M. genitalium* to prevent reinfection [12]. Partners are recommended to abstain from sexual intercourse until adequate treatment is completed and symptoms resolve if initially present [12]. There is no specific evidence regarding the utility of condom use in these circumstances.

### 3.3. Long-Term Sequelae of* M. genitalium* Infection

The long-term reproductive consequences of* M. genitalium* infection have not been clearly determined. However, the association with PID indicates that infertility, chronic pelvic pain, and risk of ectopic pregnancy may be potential sequelae of infection with this pathogen like for* C. trachomatis* and* N. gonorrhoeae* infection [64]. This may be another argument for screening in certain populations.  Table 2 summarizes the studies that investigated the association between* M. genitalium* and infertility.* M. genitalium* can persist for months or years in infected individuals [65]. In a recent meta-analysis, it had been reported that women carrying* M. genitalium* infection are usually asymptomatic with reported estimated clearance rate of 15 months based on a large London study [24]. Despite spontaneous clearance, chronic infection may lead to tissue damage prior to clearance causing long-term health problems. Reinfection due to the partner's carrier state may also lead to reinfection leading to more chronic infection. The PID Evaluation and Clinical Health (PEACH) Study is a multicenter, randomized prospective clinical trial, the largest treatment trial of mild to moderate acute PID in the United States, involving 586 women in several centers in North America who presented with signs and symptoms of PID [41]. This study showed higher rates of infertility (22%), chronic pelvic pain (42%), and recurrent PID (31%) among women in whom* M. genitalium* had been detected on endometrial samples by PCR compared to women testing negative, but these findings were not statistically significant [15, 41]. It is unclear whether the increased risk of other infections such as chlamydia or gonorrhea lead to infertility or if* M. genitalium* itself primarily leads to infertility. Given that untreated PID can lead to long-term adverse reproductive outcomes,* M. genitalium* may contribute to adverse effects on the reproductive tract. One prospective study identified strong* M. genitalium* antibody responses among women with a diagnosis of infertility that were asymptomatic, suggesting an adverse effect of* M. genitalium* on fertility [33]. Another prospective study showed that fertile women were less likely to have PCR-proven* M. genitalium* infection compared to women with idiopathic infertility (4.4% versus 29.2%, *P* = 0.0479) [34]. Consequently, some authors would recommend screening for* M. genitalium* as part of the STI work-up given possible adverse effects such as infertility, chronic pelvic disease, risk of ectopic pregnancy, and preterm labor as well as any other health consequence associated with PID. However, evidence regarding other reproductive sequelae is even more limited, and the few studies that have evaluated reproductive sequelae have not shown any statistically significant difference between women with and without* M. genitalium* infection [41]. A single case-control study on risk of ectopic pregnancy did not find any significant association either (OR 1.0, 95% CI 0.5–2.0) [66]. Well-powered prospective studies that control for other genital tract infections and compare* M. genitalium* cases to asymptomatic noninfected women are needed to establish the long-term reproductive consequences of chronic* M. genitalium* infection.

## 4. Conclusions and Areas for Future Research

*M. genitalium* is now increasingly recognized as a STI and has been associated with PID, endometritis, cervicitis, and HIV in women though clinical manifestations and risk factors overlap with other STIs. The availability of NAAT for PCR detection of this organism will allow further investigation into the effects of* M. genitalium* infection on long-term reproductive health outcomes such as infertility, chronic pelvic pain, ectopic pregnancy, and obstetric outcomes such as preterm deliveries. Due to antibiotic resistance patterns, alternatives to azithromycin and moxifloxacin must be investigated. In the interim, clinicians should consider testing for and treating* M. genitalium* on a case-by-case basis, particularly in women diagnosed with PID or cervicitis without clinical improvement using standard regimens.

## Figures and Tables

**Table 1:** Summary of *M. genitalium* prevalence according to various studies in women.

Source	Study design	Study population	Overall *M. genitalium* prevalence (%)
Gaydos et al. [2]	Cross-sectional study	324 women attending STI clinics in Baltimore. Detected by transcription mediated amplification from vaginal, endocervical, and urine swabs	19.2

Oakeshott et al. [8]	Prospective study	2378 sexually active female students (mean age of 21) followed up between 2004 and 2008 in London. Tested vaginal swabs by PCR	3.3

Haggerty et al. [15]	Multicenter randomized controlled prospective study, PEACH study	Stored cervical and endometrial specimens of 682 women treated with cefoxitin and doxycycline for clinically suspected PID tested by PCR	15

Clarivet et al. [25]	Cross-sectional study	743 asymptomatic women attending free and anonymous STI clinics from April to August 2009. Detected by PCR in first void urine (FVU) sample	0.1

Falk et al. [23]	Cross-sectional study	465 female STI clinic attendees (mean age of 24) in Orebro, Sweden. Tested FVU and endocervical samples by PCR	6

Hancock et al. [26]	Cross-sectional study	1090 women attending the Public Health-Seattle & Kig County STI Clinic in Seattle, WA. *M. genitalium* detected by TMA from self-obtained vaginal swabs	7.7

Bjartling et al. [27]	Cross-sectional case-control study	679 women attending a gynecological outpatient clinic from 2003 through 2008. Tested urine and vaginal swabs by PCR	2.1

Uno et al. [28]	Cross-sectional study	200 women visiting the Obstetrics and Gynecology Department in Kizawa Memorial Hospital and Jaysaki Women's Clinic in Japan. Tested cervical swabs using PCR.	6.8

Gomih-Alakija et al. [29]	Cross-sectional study	350 female sex workers aged 18–50 years in Nairobi, Kenya. Tested cervical samples by TMA	12.9

Bradshaw et al. [30]	Prospective study	313 women attending Melbourne Sexual Health Center, Australia, between March 2005 and November 2007 with cervicitis/pelvic inflammatory disease and sexual contacts of proven *M. genitalium, *infected partners. Cervical, vaginal swabs, or FVU samples analyzed by PCR	10

Andersen et al. [31]	Cross-sectional study	921 women aged 21–23 provided self-collected vaginal samples by PCR	2.3

**Table 2:** Summary of studies regarding *M. genitalium* and female infertility.

Source	Study design	Study population	Findings
Clausen et al. [32]	Cross-sectional study	308 women undergoing IVF treatment in Aarhus, Denmark	*M. genitalium* was detected in 22% of women with tubal factor infertility (TFI) versus 6.3% in women without TFI

Tosh et al. [19]	Multicenter (North America) randomized controlled prospective study, PEACH study	Stored cervical and endometrial specimens of 682 women treated with cefoxitin and doxycycline for clinically suspected PID	*M. genitalium* was associated with baseline endometritis (AOR 3.0, 95% CI 1.5 to 6.1). Nonsignificant trend towards increased infertility, chronic pelvic pain and recurrent PID, decreased pregnancy, and live birth were found in this study.

Svenstrup et al. [33]	Prospective study	212 couples attending a fertility clinic in Horsens-Brædstrup or the Holstebro fertility clinic in Denmark	*M. genitalium* was found to be independently associated with TFI (AOR 4.5, 95% CI 1.2–15.6)

Grześko et al. [34]	Prospective study	51 patients with primary infertility (24 women with idiopathic infertility) and 23 women with proven fertility	*M. genitalium* was found in 19.6% of all infertile women and 4.4% of fertile women (*P* = 0.156); 29.2% among women with idiopathic infertility versus 4.4% in fertile women (*P* = 0.0479)
